# Chronological Lifespan in Yeast Is Dependent on the Accumulation of Storage Carbohydrates Mediated by Yak1, Mck1 and Rim15 Kinases

**DOI:** 10.1371/journal.pgen.1006458

**Published:** 2016-12-06

**Authors:** Lu Cao, Yingzhi Tang, Zhenzhen Quan, Zhe Zhang, Stephen G. Oliver, Nianshu Zhang

**Affiliations:** Cambridge Systems Biology Centre and Department of Biochemistry, University of Cambridge, Sanger Building, United Kingdom; Department of Neurology and Biochemistry and Molecular Biology, The John T. Macdonald Foundation Center for Medical Genetics, University of Miami School of Medicine, UNITED STATES

## Abstract

Upon starvation for glucose or any other macronutrient, yeast cells exit from the mitotic cell cycle and acquire a set of characteristics that are specific to quiescent cells to ensure longevity. Little is known about the molecular determinants that orchestrate quiescence entry and lifespan extension. Using starvation-specific gene reporters, we screened a subset of the yeast deletion library representing the genes encoding ‘signaling’ proteins. Apart from the previously characterised Rim15, Mck1 and Yak1 kinases, the SNF1/AMPK complex, the cell wall integrity pathway and a number of cell cycle regulators were shown to be necessary for proper quiescence establishment and for extension of chronological lifespan (CLS), suggesting that entry into quiescence requires the integration of starvation signals transmitted via multiple signaling pathways. The CLS of these signaling mutants, and those of the single, double and triple mutants of *RIM15*, *YAK1* and *MCK1* correlates well with the amount of storage carbohydrates but poorly with transition-phase cell cycle status. Combined removal of the glycogen and trehalose biosynthetic genes, especially *GSY2* and *TPS1*, nearly abolishes the accumulation of storage carbohydrates and severely reduces CLS. Concurrent overexpression of *GSY2* and *TSL1* or supplementation of trehalose to the growth medium ameliorates the severe CLS defects displayed by the signaling mutants (*rim15Δyak1Δ* or *rim15Δmck1Δ*). Furthermore, we reveal that the levels of intracellular reactive oxygen species are cooperatively controlled by Yak1, Rim15 and Mck1, and the three kinases mediate the *TOR1*-regulated accumulation of storage carbohydrates and CLS extension. Our data support the hypothesis that metabolic reprogramming to accumulate energy stores and the activation of anti-oxidant defence systems are coordinated by Yak1, Rim15 and Mck1 kinases to ensure quiescence entry and lifespan extension in yeast.

## Author Summary

Calorie restriction improves healthy lifespan in all the organisms from yeast to mammals. However, our knowledge of the underlying mechanisms is still limited. The budding yeast naturally experiences calorie restriction when glucose in the rich medium is exhausted and cells start to consume the fermentation products before entering the stationary phase. Here, we demonstrate that the key for yeast cells to survive in the stationary phase is to accumulate sufficient storage carbohydrates (both trehalose and glycogen) during the transition phases. This accumulation requires the integration of starvation signals tranduced from multiple signaling pathways, including those activated by the inhibition of the major nutrient sensors (TOR/PKA), the energy-sensing complex (SNF1/AMPK), and the cell wall integrity pathway. We have also shown that the levels of intracellular reactive oxygen species and the population size are limited by those starvation signals downstream of TOR. Therefore, our data suggest that metabolic reprogramming to increase energy stores, anti-oxidant defence systems and population control are cooperatively activated by a set of key signaling proteins to ensure longevity in eukaryotic cells.

## Introduction

Studies in invertebrates and rodents have consistently shown that dietary intervention mimicking calorie restriction (CR) or mutations in nutrient and growth signalling pathways can increase longevity by 30–50%, accompanied by reduced or delayed morbidity in most cases [1]. Among the nutrient signalling pathways, the conserved TOR pathway regulates lifespan from simple eukaryotes to mammals [2–3]. Active signalling through TOR limits lifespan in budding yeast, nematodes, fruit flies, and mice [3]. Feeding mice with the TOR inhibitor rapamycin, even late in life, extends lifespan, representing the first pharmacological anti-aging regime in mammals [4]. However, rapamycin treatment has many side effects, including metabolic dysregulation, proliferative defects in the hematopoietic lineage, and others [5]. The TOR pathway controls many aspects of cell physiology, promoting ribosome biogenesis, translation and thus cell growth and proliferation, but also inhibiting autophagy and the stress response [6–7]. It remains unclear which aspects of TOR signalling contribute most significantly or specifically to aging and lifespan regulation [8].

In budding yeast, lifespan is classified as replicative lifespan (RLS), defined as the number of daughter cells a mother can produce before senescence, and chronological lifespan (CLS), defined as the period of time that non-dividing cells remain alive in stationary phase [9]. Down-regulation of TOR activity extends both RLS and CLS. Deletion of the Sch9 kinase, the yeast homolog of mammalian S6K1 and a downstream effector of the TORC1 complex [10], significantly extends CLS [11]. Two mechanisms, probably acting in parallel, have been proposed to account for CLS extension in yeast cells with compromised TOR. First, *TOR1* deletion enhances mitochondrial respiration. Mitochondrial ROS produced in exponentially-growing *tor1Δ* or *sch9Δ* cells provides an adaptive hormetic signal to activate the stress response dependent on Msn2/4 and Gis1, resulting in reduced levels of ROS in stationary phase cells and their elevated survival [12]. This mtROS-activated hormesis and longevity extension also involves Rph1-dependent epigenetic silencing at subtelomeric heterochromatin *via* Tel1 and Rad53, homologs of the mammalian DNA damage response kinases ATM and Chk2 [13]. It is proposed that activation of mitochondrial respiration above a threshold level to accumulate sufficient nutrient stores (storage carbohydrates) is essential to stress resistance and CLS extension [14].

CLS extension as a result of *tor1Δ* or *sch9Δ* deletion is also dependent on the Rim15 kinase (analogous to the Greatwall kinase) and its downstream effectors, Msn2/4 and Gis1, to activate stress response [15]. Nutrient starvation or TORC1 inhibition activates Rim15 [16], which in turn, via the yeast endosulfines Igo1/Igo2, prevents newly expressed mRNAs from decapping and degradation [17] and preserves its effectors in a phosphorylated (active) state by inhibiting PP2A^Cdc55^ phosphatase activity [18]. A few lines of evidence suggest that other aspects of the nutrient-sensing pathways are involved in CLS regulation. Firstly, in response to severe calorie restriction (CR), the yeast Fkh1 and Fkh2, orthologs of metazoan FOXO transcription factors, regulate chronological lifespan and oxidative stress response together with the anaphase-promoting complex [19]. How nutrient starvation signals are transmitted to Fkh1 and Fkh2 is not clear. Secondly, we have identified Yak1, the yeast homolog of the mammalian DYRKs, as the kinase acting in parallel with Rim15 to activate stress response dependent on Msn2/4 and Gis1 in TORC1-inhibited cells [20]. Recently, we have shown that the yeast GSK-3 homolog Mck1 is a key regulator of quiescence entry and Mck1 acts in parallel to Rim15 to activate starvation-induced gene expression, the acquisition of stress resistance, the accumulation of storage carbohydrates, and the extension of CLS [21]. Mck1 is necessary to regulate ribosome and tRNA synthesis [22] and to mediate very long chain fatty acid synthesis and autophagy [23] in TORC1-inhibited cells, suggesting that the function of Mck1 may be regulated by the TOR pathway. These studies indicate that starvation-induced stress response, one of the major mechanisms of modulating CLS, involves a more complex signalling network than previously thought. Here, using reporters whose expression is induced by starvation, we screened a subset of the deletion library representing the ‘signaling’ mutants and revealed that the stress response, quiescence establishment and CLS extension require the integration of multiple signals, including those transduced from the TOR/PKA, SNF1/AMPK and the cell wall integrity (CWI) pathways. We demonstrated that starvation-induced stress resistance and CLS extension in WT or *tor1Δ* cells is dependent on the accumulation of both glycogen and trehalose, mediated by the Rim15, Yak1 and Mck1 kinases. The three kinases also cooperate to control the levels of intracellular reactive oxygen species as well as the population entering the stationary phase, therefore increasing the storage carbohydrates and the capacity to defend against oxidative stress in single cells. These findings suggest that metabolic reprogramming to increase energy stores and the activation of the anti-oxidant defence systems are the primary objectives of the anti-aging signaling network at a time of nutrient scarcity.

## Results

### The SNF1/AMPK and the cell wall integrity (CWI) pathways are necessary for starvation-induced gene expression and CLS extension

**Fig 1:**
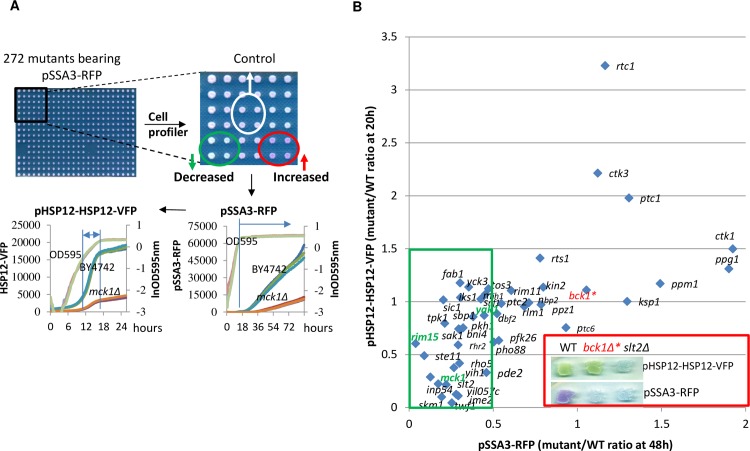
Identifying signaling mutants displaying defects in starvation-induced gene expression. **1A:** 272 signalling mutants and WT cells transformed with the pSSA3-RFP construct were arrayed in quadruplets on SMM medium containing 0.6% glucose and supplements, grown for 2–3 days. Images with a dark background obtained by scanning were analysed with Cell Profiler (http://www.cellprofiler.org/). Those showing significant increased or decreased expression of pSSA3-RFP were subjected to quantitative assay of both reporters and cell growth (OD_595nm_) using a plate reader. **1B:** Relative expressions of pSSA3-RFP and pHSP12-HSP12-VFP in mutants to those in WT cells after normalising to OD_595nm_. Contained in the red box are WT, *bck1Δ* and *slt2Δ* deletion cells bearing the pHSP12-HSP12-VFP or pSSA3-RFP constructs, patched on SMM (agar) medium and grown for 2 days before imaging.

Wei *et al*. [15] have predicted that other factors than Rim15 are involved in regulating the stress response and lifespan extension by calorie restriction. To facilitate the automated identification of these factors regulating the stress response, the deletion mutants of the 272 genes in the yeast genome that encode the majority of the ‘signaling’ molecules were screened using two starvation-induced gene reporters, pHSP12-HSP12-VFP and pSSA3-RFP, following the procedures described in Fig 1A. Starvation-induced expression of the two reporters have been shown to be dependent on Msn2/4 and Gis1 [21]. In the *SSA3* promoter, we also identified a heat shock element (HSE, 5’-NGAANN_5_NGAANN_5_NGAAN-3’), which is targeted by the heat shock factor, Hsf1 [24]. Decreasing the level of Hsf1 by using a DAmP strain [25] or mutating the HSE in the *SSA3* promoter (see Materials and Methods) significantly reduced the levels of RFP (S1A and S1B Fig), suggesting that pSSA3-RFP expression induced by starvation is also regulated by Hsf1. Mutating the HSE in the *SSA3* promoter completely abolished the expression of RFP in the *msn2/4Δgis1Δ* mutants (S1B Fig). These data confirmed previous observations that Hsf1, Msn2/4 and Gis1 cooperate to regulate starvation-induced stress response [20,26].

In comparison to wild-type cells, 45 mutants (about 16%) displayed significant changes of pSSA3-RFP and/or pHSP12-HSP12-VFP levels (Fig 1B, p<0.05). The negative regulators, exhibiting higher levels of the fluorescent reporters in their mutants (Fig 1B), are subunits of C-terminal domain kinase I (*CTK1* and *CTK3*), components of PP2A complex (*PPM1* and *RTS1*), PP2A-like phosphatase (*PPG1*) and PP2C (*PTC1*). In contrast, besides the previously reported *RIM15*, *YAK1*, and *MCK1* (see the introduction), deletion of genes involved in a number of pathways caused a significant decrease of starvation-induced gene expression. These include genes implicated in the cell wall integrity pathway (*PKH1*, *RHO5*, *BCK1*, *SLT2* and *RLM1*), in the SNF1/AMPK complex (*SNF1*, *TOS3* and *SAK1*), in the PKA (*PDE2* and *TPK1*) and the MAPK pathway governing meiosis (*IME2* and *STE11*), in cell cycle control (*SKM1*, *SIC1* and *MIH1*), and a couple of kinases whose expression is induced by mild-heat stress (*IKS1*) or by carbon limitation (*RGI2*). As compared to WT cells, the expression of pSSA3-RFP in the *bck1Δ* mutants was induced to a similar level in the first 48 hours (Fig 1B) but its accumulation was severely decreased, to a level similar to that in the *slt2Δ* cells when assayed on agar plates (see the red box in Fig 1B). Many more mutants (37) displayed decreased expression of the reporters than those (8) showing increased levels (Fig 1B), suggesting that activation of starvation-specific gene expression may involve the integration of signals transduced by multiple pathways.

**Fig 2:**
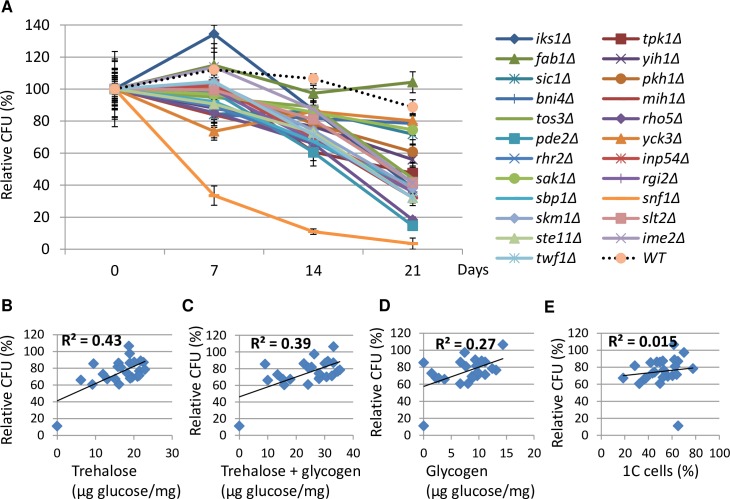
CLS of the signaling mutants is well correlated with the amount of storage carbohydrates. **2A:** Relative CFU of WT and ‘signaling’ mutants at 7, 14 and 21 days normalised to that at day 0. Day 0 data is collected after cells were grown in YPD for three days to early stationary phase. **2B, 2C, 2D and 2E:** Correlation coefficient (R) between relative CFU (at day 14) and trehalose (2B), the sum of trehalose and glycogen (2C), glycogen (2D) or the ratio of G_d_/G_1_ (1C) cells at 24h (2E).

Apart from *RIM15*, *YAK1*, and *MCK1* (see next section), deletion of 23 genes led to a severe decrease of pSSA3-RFP expression (>2-fold, boxed in green in Fig 1B). To discover whether these mutants displayed other defects associated with the transition phases, we also analyzed their FACS profiles at the late-exponential phase (11h) and during the transition to stationary phase (24h; Table 1), the accumulation of glycogen and trehalose at early stationary phase (72h, Table 1) and their ability to exit from quiescence during the stationary phase (Fig 2A). As compared to WT cells, a number of mutants, including *bni4Δ*, *rho5Δ*, *rhr2Δ*, *sic1Δ* and *pde2Δ*, exhibited a significantly higher proportion of budded (S/G_2_/M) cells at both late exponential and transition phases (compare 11h and 24h, Table 1), suggesting defects in G_2_/M transition or mitosis. The high budding index displayed by the *slt2Δ* or *yck3Δ* mutant at the late exponential phase decreased in the transition phase, whereas those of *sbp1Δ*, *sak1Δ* and *ste11Δ* increased dramatically, indicating that these signaling proteins regulate the cell cycle progression in a way dependent on the nutrient status. Previous cell sorting and FACS analyses [21] revealed three distinct populations in WT cell cultures in transition phases: small daughter cells (G_d_), larger daughter or unbudded mother cells (G_1_) and the budded cells (S/G_2_/M, S2 Fig). In the transition-phase cultures of *sbp1Δ*, *sak1Δ* or *rhr2Δ* mutants, an extra population representing a 3C DNA content was evident from their FACS profiles (encircled in red, S2 Fig), suggesting defects of cytokinesis or cell separation when these mutants were growing on non-fermentable carbon sources. The majority of these signaling mutants showed a reduced CFU when compared to WT cells over a period of 21 days (Fig 2A). The relative CFU (at day 14) of these mutants seems to correlate well with the amount of trehalose (R^2^ = 0.43; Fig 2B) or the total amount of trehalose and glycogen (R^2^ = 0.39, Fig 2C), less well with glycogen (R^2^ = 0.27, Fig 2D), but very poorly with the ratio of 1C cells (G_d_/G_1_) in the transition phase (R^2^ = 0.015; Fig 2E), suggesting that signalling to accumulate energy stores, especially trehalose, may be important to long-term survival.

**Table 1:** Budding index and storage carbohydrates accumulated in the transition phase.

Name	Functional category	Gene function	S-G2-M cells (%)		Glycogen (μg/mg, 72h)	Trehalose (μg/mg, 72h)
			11h	24h	mean ±stdev	mean ±stdev
WT	−	−	40.9	34.5	10.0 ± 0.2	23.2 ± 1.1
*PKH1*	Cell wall integrity	Serine/threonine protein kinase	32	22.4	12.4 ± 0.7	22.9 ± 0.3
*RHO5*		Non-essential small GTPase	75.3	63.7	3.8 ± 0.2	6.2 ± 0.7
*SLT2*		Serine/threonine MAP kinase	75.6	37.5	7.7 ± 0.5	16.6 ± 0.1
*TOS3*	SNF1 complex	Protein kinase for the activation of Snf1p	54.7	38	9.3 ± 0.3	21.8 ± 1.6
*SAK1*		Upstream kinase for the SNF1 complex	37.7	58.1	0.5 ± 0.1	9.5 ± 1.7
*SNF1*		AMP-activated S/T protein kinase	46.1	34.9	0.8 ± 0.1	0.6 ± 0.1
*PDE2*	PKA pathway	High-affinity cyclic AMP phosphodiesterase	63.4	67.7	6.6 ± 1.2	9.2 ± 0.4
*TPK1*		cAMP-dependent protein kinase catalytic subunit	48.8	50.3	8.0 ± 0.1	16.2 ± 0.7
*SKM1*	Cell cycle cytoskeleton	Member of the PAK family of serine/threonine protein kinases	38.7	54.7	9.8 ± 0.3	18.3 ± 1.4
*SIC1*		Cyclin-dependent kinase inhibitor (CKI)	65.6	53	11.1 ± 0.1	19.0 ± 0.6
*MIH1*		Protein tyrosine phosphatase	36.3	43.6	8.8 ± 0.3	19.5 ± 1.5
*TWF1*		Twinfilin	34.3	36.2	11.1 ± 0.4	21.4 ± 1.5
*BNI4*		Targeting subunit for Glc7p protein phosphatase	69.4	71.5	9.6 ± 0.1	15.8 ± 0.9
*STE11*	MAPK pathway	Signal transducing MEK kinase	36.7	56.6	1.5 ± 0.3	12.0 ± 1.3
*IME2*		Serine/threonine protein kinase involved in activation of meiosis	38.5	47.8	10.5 ± 0.2	20.1 ± 0.4
*RHR2*	Glycerol biosynthesis	Constitutively expressed DL-glycerol-3-phosphate phosphatase	70.5	81.4	2.3 ± 0.1	13.1 ± 0.7
*INP54*	Secretion, vacuole sorting and fusion	Phosphatidylinositol 4,5-bisphosphate 5-phosphatase	32.3	39.7	9.7 ± 0.3	21.0 ± 0.7
*YCK3*		Palmitoylated vacuolar membrane-localized casein kinase I isoform	79.4	50.2	6.7 ± 1	16.0 ± 0.4
*FAB1*		1-phosphatidylinositol-3-phosphate 5-kinase	51.4	30	7.4 ± 0.3	18.9 ± 0.3
*YIH1*	Regulation of translation in response to glucose starvation	Negative regulator of eIF2 kinase Gcn2p	53.5	47	13.1 ± 1.5	21.4 ± 2.0
*SBP1*		Protein that binds eIF4G and has a role in repression of translation	40.6	62.1	2.7 ± 0.2	15.2 ± 0.9
*RGI2*	Unknown	Protein of unknown function	48.5	47.7	8.9 ± 0.4	19.1 ± 1
*IKS1*		Protein kinase of unknown cellular role	32.5	35	10.6 ± 0.5	22.5 ± 1.5

### CLS of the single, double and triple mutants of *YAK1*, *MCK1* and *RIM15* is strongly correlated with the levels of storage carbohydrates

**Fig 3:**
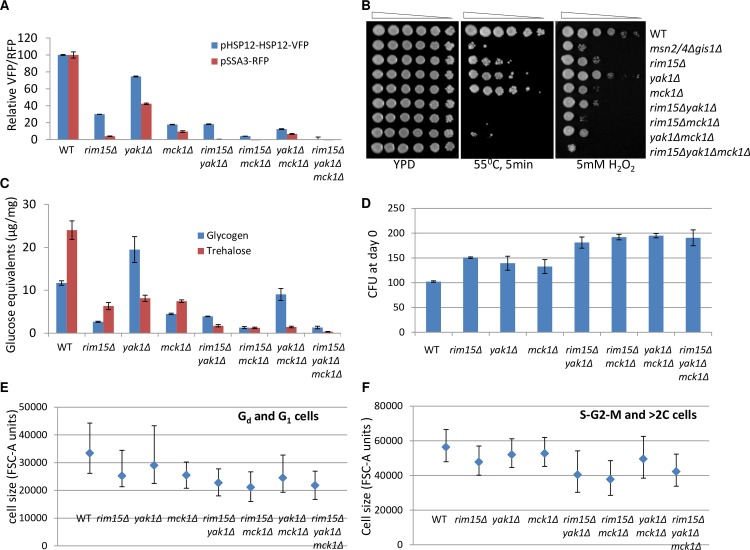
Phenotypic analysis of the single, double and triple mutants of *RIM15*, *YAK1* and *MCK1*. **3A:** Expression levels of pHSP12-HSP12-VFP at 20h and of pSSA3-RFP at 48h in the above mutants. **3B:** Heat and oxidative stress resistance displayed by WT and mutant cells grown for 3 days in YPD. **3C:** Glycogen and trehalose accumulated in WT and mutant cells grown for 3 days in YPD. **3D:** The number of colonies formed by WT and mutant cell cultures grown in YPD for 3 days. **3E:** Median, 1^st^ and 3^rd^ quartiles of cell size of the G_d_ and G_1_ populations in the day 3 culture. **3F:** Median, 1^st^ and 3^rd^ quartiles of cell size of the S-G_2_-M and >2C populations in the day 3 culture. At least 100,000 G_d_/G_1_ and 15,000 ≥2C cells from each culture were scored. The pairwise comparisons between WT and each mutant in 3e and 3f reveal that all p values calculated from the t-test scores are less than 0.0001.

Many of the yeast proteins implicated in signaling pathways that are required to activate starvation-induced gene expression have mammalian orthologs. Yak1 belongs to the group of dual-specificity tyrosine phosphorylation-regulated kinases (DYRKs)[27]. Upon TORC1 inhibition, Yak1 translocates to the nucleus to activate the transcription of Msn2 /4 targets [20,28] and to down-regulate the expression of ribosomal protein genes [29]. The GSK-1 homolog Mck1 was recently shown to act in parallel to the Rim15 kinase to enable the acquisition of a number of quiescence-related characteristics and CLS extension [21]. Previously, *PDR5* was removed from WT, and the *rim15Δ*, *mck1Δ* and *rim15Δmck1Δ* mutants in order to find how the proteasome function influences starvation-induced gene expression [21]. To reveal the genetic relationship between *YAK1* and *RIM15* or *MCK1* in starvation-induced stress response, the levels of pHSP12-HSP12-VFP and pSSA3-RFP reporters were determined in the single, double and triple mutants of *YAK1*, *RIM15* and *MCK1*, which were generated in the same genetic background (*pdr5Δ*). The *rim15Δmck1Δ* double mutants were included for comparison. As compared to the single mutants, the expression of pHSP12-HSP12-VFP and pSSA3-RFP was dramatically reduced in the *rim15Δyak1Δ* or *yak1Δmck1Δ* double mutants, albeit to a lesser degree than that seen in the *rim15Δmck1Δ* deletants (Fig 3A). Deletion of all three genes completely abolished the expression of both reporters (Fig 3A). These data suggest that Yak1 activates starvation-induced gene expression in parallel or compensatory pathways to those of Rim15 and Mck1. To further examine the physiological implications of the above findings, stress resistance exhibited by early stationary-phase cells (grown in YPD for 3 days) was monitored. As shown in Fig 3B, the *yak1Δ* single mutants display severe defects in response to heat shock and moderate sensitivity to oxidative stress as compared to WT cells. The *rim15Δyak1Δ* or *yak1Δmck1Δ* double mutants, similar to the *rim15Δmck1Δ* mutants, recapitulate the heat shock sensitivity of the *gis1Δmsn2/4Δ* triple mutants and display stronger defects in response to oxidative stress than the latter, indicating that the three kinases cooperate to control the acquisition of stress resistance during the transition into stationary phase.

The cellular content of storage carbohydrates (trehalose and glycogen) was also determined after 3 days in YPD. As compared to that observed in wild-type cells, accumulation of trehalose was severely reduced in the *yak1Δ* mutants (Fig 3C). In mutants bearing double deletions of *YAK1* and *RIM15* or *YAK1* and *MCK1*, the amount of trehalose was lower than that in the single kinase mutants, similar to what was seen in the *rim15Δmck1Δ* mutants. The accumulation of trehalose was almost abolished in the *rim15Δyak1Δmck1Δ* triple mutant (Fig 3C), indicating that the three kinases may act in parallel and/or compensatory pathways to enable trehalose accumulation. In contrast to the severe decrease of glycogen seen in the *rim15Δ* or *mck1Δ* deletion cells, deletion of *YAK1* led to a dramatic increase of glycogen (Fig 3C), a phenotype which has been reported previously (Wilson et al., 2002). *YAK1* deletion led to a significant increase of glycogen in the *mck1Δ* cells but only a marginal increase in the *rim15Δ* or *rim15Δmck1Δ* mutants (Fig 3C), suggesting that glycogen hyper-accumulation due to *YAK1* deletion is predominantly mediated through the Rim15 pathway.

After 3 days of growth in YPD, the number of colonies formed by the *yak1Δ* cell culture was increased by ~40% compared to that by the WT culture (Fig 3D). A similar increase was also seen from those produced by the *rim15Δ* or *mck1Δ* mutants (Fig 3D). Further deletion of *RIM15* or *MCK1* from the *yak1Δ* mutant increased the CFU by ~100%, to a similar extent to that formed by the *rim15Δmck1Δ* double mutant (Fig 3D), suggesting that Yak1 acts together with Rim15 or Mck1 to limit the population entering the stationary phase. Cell division in budding yeast is extremely asymmetric under nutrient limitations (Lord and Wheals, 1981). We have previously demonstrated that, in the WT cell culture, FACS can be used to differentiate the new daughter cells (G_d_) from those having grown to the size of their mothers (G_1_) and those having entered into the S phase and mitosis (S-G_2_-M cells) ([21], S2 and S3 Figs). In the *rim15Δmck1Δ* double mutant culture, the G_1_ population cannot be discriminated from G_d_ cells by FACS [21] and the size of G_d_/G_1_ or S-G_2_-M cells is significantly decreased (Fig 3E and 3F), indicating that Mck1 and Rim15 function to maintain the size threshold at the G_1_/S transition to restrict the population growth. The median and size distribution of the G_d_/G_1_ or S-G_2_-M cells in the *yak1Δ* culture is moderately reduced as compared to those in the WT culture (Fig 3E and 3F). Removal of *YAK1* from the *rim15Δ* mutant leads to even smaller G_d_/G_1_ or S-G_2_-M cells (Fig 3E and 3F) and coalescence of the two populations in the FACS profile (S3 Fig), similar to what was seen in the *rim15Δmck1Δ* mutants (Fig 3E and [21]). Deletion of *YAK1* from the *mck1Δ* mutant marginally reduces the size of G_d_/G_1_ or S-G_2_-M cells (Fig 3E and 3F) but significantly decreases the >2C population observed in the *mck1Δ* culture (S3 Fig). These data suggest that Yak1 may act together with Rim15 to regulate the size threshold for the G_1_/S transition to control the population growth. The population increase seen in the *yak1Δmck1Δ* culture may be due to a combination of size control defects (Fig 3E) and the resolving of the multi-budded population (S3 Fig). These data suggest that Yak1, Mck1 and Rim15 kinases cooperate to regulate the accumulation of storage carbohydrates and the population entering the stationary phase.

**Fig 4:**
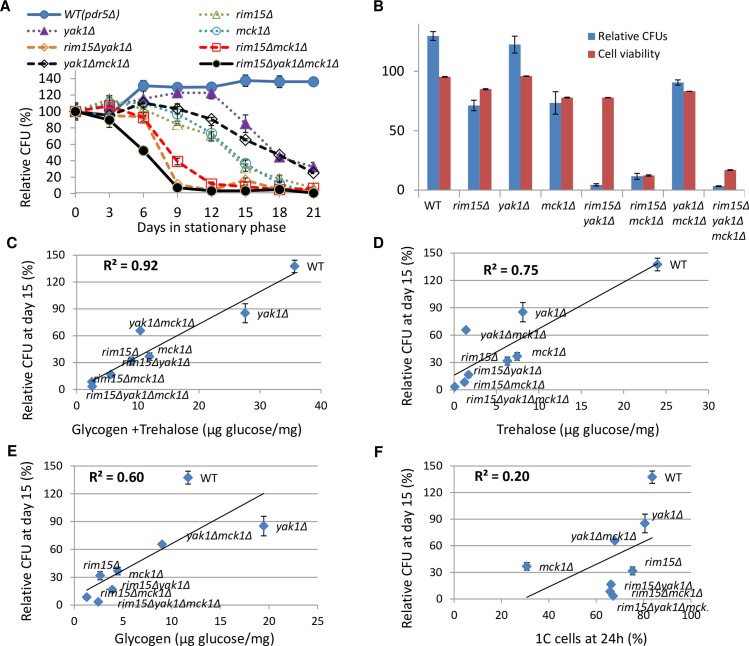
Chronological lifespan of the single, double and triple mutants of *RIM15*, *YAK1* and *MCK1* is well correlated with the accumulation of storage carbohydrates. **4A:** Relative CFU measured over 21 days. **4B:** Comparison between relative CFU and cell survival displayed by WT and mutant cultures at day 12. **4C**, **4D** and **4E:** Correlation between relative CFU (at day 15) and total amount of storage carbohydrates (4C), trehalose (4D) or glycogen (4E). **4F:** Correlation between relative CFU and the ratio of G_d_/G_1_ (1C) cells at 24h in YPD.

To determine CLS, the CFU of the WT and mutant cell cultures were monitored for a further 21 days and normalised to that formed by day 0 culture (grown in YPD for 3 days in Fig 4). The pH values of the spent media from both WT and mutant cultures were around 4.5, ruling out the complication of medium acidification reducing lifespan [30]. The *yak1Δ* mutant exhibited a lower relative CFU than WT cells only at the late stationary phase, contrasting to the earlier decrease seen in the *rim15Δ* or *mck1Δ* mutants (Fig 4A). Removal of *YAK1* from the *rim15Δ* cells (*rim15Δyak1Δ*) dramatically decreased the ability of stationary-phase cells to form colonies, to an extent similar to that observed for the *rim15Δmck1Δ* double mutants (Fig 4A). Cell viability of the *rim15Δyak1Δ* mutants, however, is only marginally lower than that of the *rim15Δ* cells (Fig 4B). This is in stark contrast to the dramatic loss of cell survival in the *rim15Δmck1Δ* culture (Fig 4B and [21]). Further deletion of *YAK1* from the *mck1Δ* cells (*yak1Δmck1Δ*), increased relative CFU (Fig 4A) and marginally improved cell viability (Fig 4B). These observations indicate that the Yak1 kinase regulates quiescence exit in a Rim15-dependent manner, similar to its relationship with Rim15 in glycogen accumulation. Relative CFU at day 15 of the single, double and triple mutants of *YAK1*, *RIM15* and *MCK1* correlate very well with the total amount of storage carbohydrates (R^2^ = 0.92, Fig 4C), less well with trehalose (R^2^ = 0.75, Fig 4D) or glycogen (R^2^ = 0.60, Fig 4E), but poorly with the ratio of 1C (G_d_/G_1_) cells (R^2^ = 0.20; Fig 4F). Relative CFU at day 12 or day 18 are also well correlated with the sum of trehalose and glycogen (S4A and S4B Fig). These data suggest that the accumulation of trehalose plays a more dominant role than glycogen in quiescence exit, but the storage of both energy stores is necessary for CLS extension.

### The ability to exit from quiescence is dependent on both trehalose and glycogen

**Fig 5:**
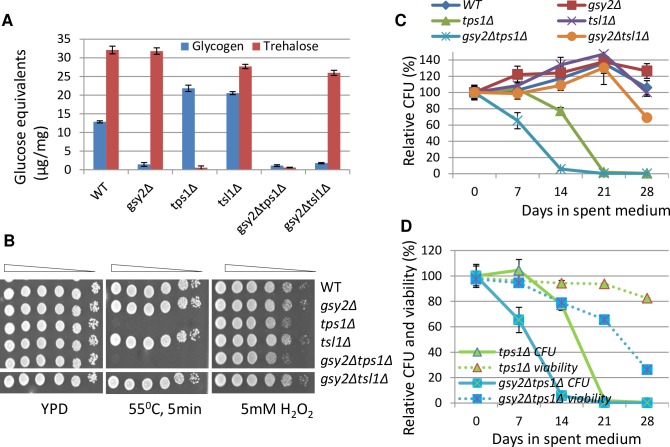
The accumulation of both trehalose and glycogen is necessary for quiescence exit. **5A:** Glycogen and trehalose levels determined in the *gsy2Δ*, *tps1Δ* and *tsl1Δ* single and the *gsy2Δtps1Δ* and *gsy2tsl1Δ* double mutants. **5B:** Heat and oxidative stress resistance displayed by the above mutants. **5C:** Relative CFU of the above mutants over 28 days. **5D:** Comparison between relative CFU and cell viability of the *tps1Δ* and *gsy2Δtps1Δ* mutants over 28 days.

To confirm that the accumulation of both trehalose and glycogen are necessary for exit from quiescence, *GSY2*, responsible for the majority (~90%) of the glycogen synthase activity in *S*. *cerevisiae* [31], was removed from the *tps1Δ* or *tsl1Δ* mutants. *TPS1* encodes the synthase subunit of trehalose-6-P synthase/phosphatase (TPS/TPP) complex and its deletion results in loss of both TPS activity and trehalose biosynthesis [32]. *TSL1* encodes a regulatory protein for TPS/TPP and removal of *TSL1* has only minor effects on TPS/TPP activities and on trehalose biosynthesis [33]. The levels of trehalose and glycogen accumulated in WT and mutant cells were consistent with these observations (Fig 5A). The accumulation of trehalose was not changed in the *gsy2Δ* mutants, whereas a great increase of glycogen was seen in the *tps1Δ* cells (Fig 5A), suggesting that biosynthetic activity was directed towards glycogen when trehalose biosynthesis was compromised. Profound sensitivity to heat or mild sensitivity to oxidative stress was associated with *tps1Δ*, independent of *GSY2* (Fig 5B); this agrees with previous observations that *tps1Δ* mutants showed hypersensitivity to various stresses [34–40]. Relative CFU formed by the stationary-phase *tps1Δ* cells was reduced to less than 5% by day 21, whereas those produced by the *gsy2Δ* or *tsl1Δ* deletants remained similar to that of the WT cells (Fig 5C). Further deletion of *GSY2* from the *tps1Δ* cells (*gsy2Δtps1Δ*) exacerbates its CFU defects (Fig 5C). Deletion of *GSY2* from the *tsl1Δ* mutant (*gsy2Δtsl1Δ*) also results in a mild CFU defect which was not observed in *gsy2Δ* or *tsl1Δ* single mutant (Fig 5C). Relative CFU at day 14 among WT and these mutants correlates poorly with glycogen (R^2^ = 0.09, S4C Fig), very well with trehalose (R^2^ = 0.73, S4D Fig), and best with the total amount of storage carbohydrates (R^2^ = 0.88, S4E Fig). The majority of the *tps1Δ* or *gsy2Δtps1Δ* cells remained viable when they lost the ability to form colonies and these cells gradually become inviable (Fig 5D), further suggesting that failure to accumulate storage carbohydrates causes defects first in quiescence exit and subsequently in cell survival. The *gsy2Δtps1Δ* cells lost their viability more quickly than the *tps1Δ* mutants (Fig 5D). Put together, these data support that the accumulation of trehalose plays a dominant role in quiescence exit but glycogen storage contributes to long-term survival.

**Fig 6:**
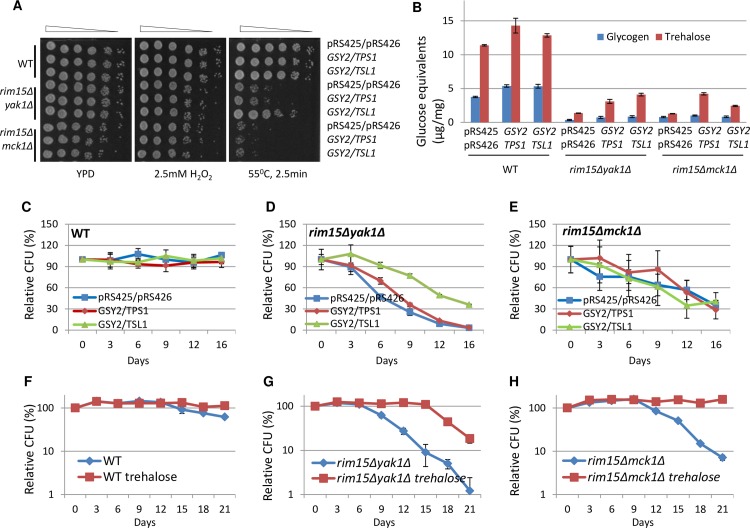
The CLS defects in the *rim15Δyak1Δ* or *rim15Δmck1Δ* mutants are rescued by *GSY2*/*TSL1* overexpression and/or trehalose supplementation. **6A** and **6B**: Stress resistance (6A) and storage carbohydrates (6B) accumulated in the *rim15Δyak1Δ* or *rim15Δmck1Δ* cells bearing the empty vectors, *GSY2*/*TPS1* or *GSY2/TSL1* overexpression plasmids. **6C**, **6D** and **6E:** Relative CFU of WT (6C), the *rim15Δyak1Δ* (6D) and *rim15Δmck1Δ* (6E) mutants overexpressing *GSY2*/*TPS1* or *GSY2/TSL1*. **6F**, **6G** and **6H:** Supplementation of trehalose enhances the ability of WT cells to exit from quiescence (6F), ameliorates or rescues the CFU defects of the *rim15Δyak1Δ* (6G) and *rim15Δmck1Δ* (6H) cells. Cells were grown in YPD supplemented with 1% Trehalose from inoculation.

The accumulation of both storage carbohydrates is substantially decreased in the *rim15Δyak1Δ* or *rim15Δmck1Δ* double mutants (Fig 3C) and they were unable to exit from quiescence during mid-stationary phase (Fig 4A). To confirm that quiescence exit regulated by Rim15 and Yak1 or Rim15 and Mck1 is dependent on the accumulation of storage carbohydrates, we attempted (but failed) to remove both *RIM15* and *YAK1* or *RIM15* and *MCK1* from the *gsy2Δtps1Δ* mutants. Instead, *GSY2* and *TPS1* or *TSL1* were overexpressed in the *rim15Δyak1Δ* or *rim15Δmck1Δ* double mutants. Cells bearing the overexpression plasmids were grown in buffered SC medium [41] to avoid the adverse effect of acidification on CLS. After growth for 3 days, cells were washed and resuspended in sterile water for CFU assays to prevent cryptic growth observed for the *rim15Δyak1Δ* double mutants (Fig 4A). Concurrent expression of *GSY2* and *TSL1* in the *rim15Δyak1Δ* cells enhanced their resistance to heat stress (Fig 6A), increased the amount of glycogen and trehalose by ~2- and 3~4-fold respectively (Fig 6B), and significantly improved their capability to form colonies (Fig 6D) and their survival rate (S5B Fig). Concurrent overexpression of *GSY2* and *TPS1* in the *rim15Δyak1Δ* cells also enhanced the levels of glycogen and trehalose, albeit to a lesser degree than those seen in the same cells overexpressing *GSY2* and *TSL1* (Fig 6B). However, heat stress resistance is not improved (Fig 6A), CFU is only marginally increased (Fig 6D) and their survival rate remained the same as the same cells bearing the empty vectors (S5B Fig). The levels of trehalose and glycogen are increased in WT cells overexpressing *GSY2*/*TPS1* or *GSY2*/*TSL1* (Fig 6B). Stress resistance (Fig 6A), relative CFU (Fig 6C) or cell survival (S5A Fig) displayed by these cells is similar to those bearing the empty vectors. These data suggest that the accumulation of storage carbohydrates above a certain threshold is essential to enable stress resistance and CLS extension.

The accumulation of glycogen was marginally and that of trehalose significantly increased in the *rim15Δmck1Δ* mutants overexpressing *GSY2* and *TPS1*, and to a lesser extent upon coordinate overexpression of *GSY2* and *TSL1* (Fig 6B). However, no enhanced stress resistance (Fig 6A) and only marginal increase of CFU (Fig 6E) was observed in the *rim15Δmck1Δ* mutants overexpressing *GSY2* and *TPS1*. On day 0, the number of colonies grown from the *rim15Δmck1Δ* culture is only around a third of those from the *rim15Δyak1Δ* or WT cultures. Viability tests revealed that the vast majority of the *rim15Δmck1Δ* cells (>60–70%) had died after three days of incubation in buffered SC medium (S5C Fig), a phenotype seen in rich medium only after 12 days (Fig 4B). Thus, deletion of *RIM15* and *MCK1* may lead to substantial cell death in poorer nutrient conditions. A moderate increase of storage carbohydrates is not sufficient to rescue this lethality.

Trehalose can be assimilated by actively-growing cells [14]. 1% trehalose was added into the growth medium from inoculation. Addition of trehalose mildly improved the CFU of WT cells in the stationary phase (Fig 6F), partially rescued the CFU defects exhibited by the *rim15Δyak1Δ* mutants (Fig 6G) and dramatically restored the relative CFU of the *rim15Δmck1Δ* mutants close to the WT levels (Fig 6H). These observations further confirmed that signaling to accumulate storage carbohydrates is crucial to CLS extension.

### Rim15, Yak1 and Mck1 cooperate to control intracellular reactive oxygen species (ROS)

**Fig 7:**
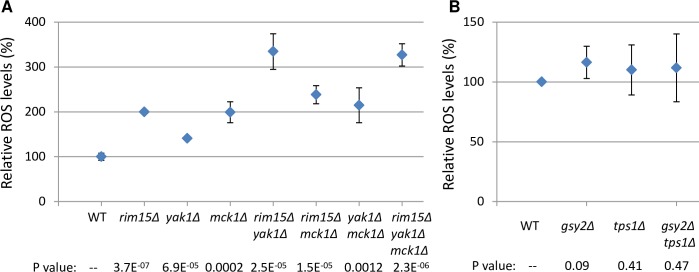
The levels of intracellular ROS in early stationary-phase cells are controlled by Rim15, Yak1 and Mck1. **7A**: ROS levels determined in WT, the single, double and triple mutants of *RIM15*, *YAK1* and *MCK1*. **7B**: ROS levels detected in WT, the single and double mutants of *GSY2* and *TPS1*. WT and mutant cells were grown in YPD for 3 days before staining by DHR. The mean value represents the average of two biological and two technical replicates. In each replicate, at least 10, 000 cells were scored. The p values, listed below each mutant, were calculated by pairwise t-test between the WT and mutant.

The accumulation of storage carbohydrates in the *rim15Δyak1Δmck1Δ* triple mutant or the *gsy2Δtps1Δ* double deletant is nearly abolished (Fig 3C and Fig 5A). However, the former lost their ability to exit from quiescence more quickly than the latter (compare Fig 4A and Fig 5C). Although both mutants displayed similar defects in resistance to heat shock (Fig 3B and Fig 5B), the *rim15Δyak1Δmck1Δ* mutants exhibited a more severe defect in their resistance to exogenous oxidant than the *gsy2Δtps1Δ* cells (Fig 3B and Fig 5B), indicating that the three kinases may cooperate to regulate the anti-oxidant defence systems to extend CLS. To confirm this, dihydrorhodamine 123 (DHR) was used to determine the levels of intracellular ROS in early stationary-phase cells (see Materials and Methods). As compared to WT, removal of any of the three kinase genes increased the levels of ROS significantly (see the p values listed below each mutant in Fig 7A). Among the three double mutants, only the *rim15Δyak1Δ* cells accumulated much higher levels of ROS than those in the *rim15Δ* or *yak1Δ* single mutants (Fig 7A), suggesting that Rim15 and Yak1 may activate the antioxidant systems in parallel to remove intracellular ROS. Removal of *MCK1* from the *rim15Δ* or the *rim15Δyak1Δ* double mutants, however, did not increase the DHR-staining signals significantly, suggesting that Mck1 plays a redundant role with Rim15 or Rim15 and Yak1 to eliminate ROS (Fig 7A). To find whether the accumulation of trehalose and/or glycogen *per se* influences the anti-oxidant defence system, intracellular ROS levels were also determined in the *gsy2Δ*, *tps1Δ* and *gsy2Δtps1Δ* cells grown to early stationary phase. As shown in Fig 7B, deletion of *GSY2*, *TPS1* or both did not greatly increase the levels of intracellular ROS. Therefore, the mild defects to resist oxidative stress in the *tps1Δ* cells (Fig 5B) could be attributed to their lack of protection from trehalose [35] rather than a defective antioxidant defence system. These data further confirm that the Rim15, Yak1 and Mck1 kinases may promote the extension of CLS by regulating the accumulation of storage carbohydrates as well as the anti-oxidant defence systems.

### *TOR1*-regulated accumulation of storage carbohydrates and CLS extension relies on Yak1, Rim15 and Mck1

**Fig 8:**
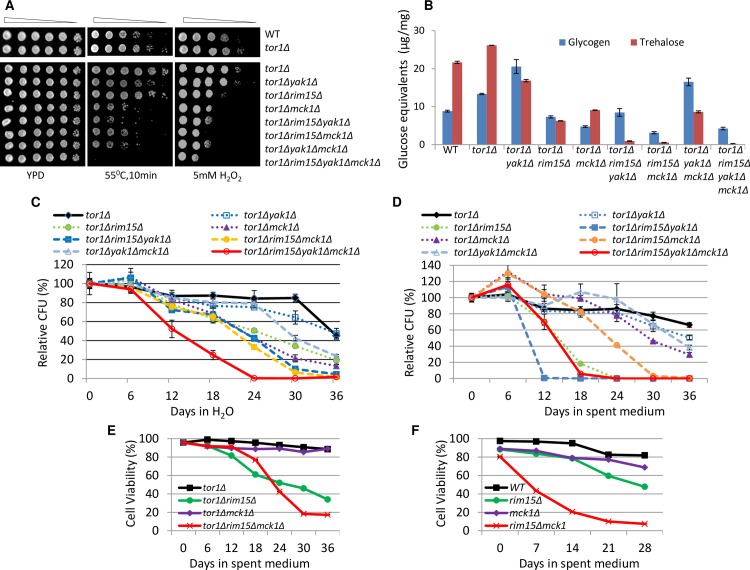
*TOR1*-regulated quiescence establishment and exit is mediated by *RIM15*, *YAK1* and *MCK1*. **8A**, **8B**, **8C** and **8D**: Stress resistance (8A), storage carbohydrates (8B), relative CFU in H_2_O (8C) or relative CFU in spent medium (8D) displayed by the *tor1Δ* mutants carrying also the single, double or triple deletions of *RIM15*, *YAK1* and *MCK1*. **8E** and **8F**: Comparison of cell survival of WT, *rim15Δ*, *mck1Δ* and *rim15Δmck1Δ* cells in spent medium carrying *tor1Δ* deletion (8E) or an intact *TOR1* (8F).

A previous study by Wei *et al*. [15] suggested that additional regulators other than Rim15 in the TOR signaling pathway are involved in mediating CLS extension. To find whether Yak1 and/or Mck1 are these additional regulators, we replaced *TOR1* with *K*. *lactis LEU2* (*KlLEU2*) in the above WT, and the single, double and triple mutants of *YAK1*, *RIM15* and *MCK1*. *KlLEU2* was also integrated at the *lys2Δ* locus to generate a wild-type strain isogenic to *tor1Δ*::*KlLEU2*. Deletion of *TOR1* from the WT cells was reported to enhance stress resistance and CLS [15,42]. Replacing *TOR1* with *KlLEU2* in BY4742 mildly increased the resistance to extended heat stress (10min at 55°C in Fig 8A). The increased resistance to heat stress conferred by *tor1Δ* is principally dependent on Mck1 among the three kinases, and on Rim15 and Yak1 among the pair-wise combinations of the three regulators (Fig 8A). Removal of all three genes abolished the resistance to heat stress (Fig 8A), indicating that all three kinases are necessary to promote heat stress resistance enabled by *tor1Δ* deletion. Removal of *TOR1* also marginally increased resistance to oxidative stress (Fig 8A). Enhanced resistance to oxidative stress is mostly dependent on Rim15 and Mck1 and further removal of Yak1 did not significantly reduce resistance to oxidative stress in the *tor1Δ* cells, similar to what was seen in the WT background (Fig 3B). Deletion of *TOR1* increased the accumulation of both trehalose and glycogen (Fig 8B). Similar to what was seen in WT cells (Fig 3C), *YAK1* deletion from the *tor1Δ* mutant increased the level of glycogen and this increase is more dependent on *RIM15* than *MCK1* (Fig 8B). The accumulation of trehalose in the *tor1Δ* cells was abolished when all three kinase genes were removed (Fig 8B), similar to what was seen in the WT background (Fig 3C).

After 3 days of growth in YPD medium, the cultures were split into two halves. One half was kept in the spent medium and the other was washed and resuspended in H_2_O. Deletion of *TOR1* improved the CFU of the WT cells in water or in the spent medium in the first 30 days (S6A and S6B Fig). In water, which was considered as a condition of extreme calorie restriction [43–44], the ability of the *tor1Δ* mutant to form colonies is strongly decreased by deletion of *RIM15* or *MCK1*, and to a lesser degree, by removal of *YAK1* (Fig 8C). A further decrease of CFU, especially in the later stages of the assay (18 to 36 days), was seen when both *RIM15* and *YAK1* or *RIM15* and *MCK1* were removed (Fig 8C). In contrast, further deletion of *YAK1* from the *tor1Δmck1Δ* mutant increased their ability to form colonies (Fig 8C), similar to what was observed when *YAK1* was eliminated from the *mck1Δ* cells (Fig 4A) and consistent with the levels of storage carbohydrates accumulated during the transition phase (Fig 8B). Removal of all three genes dramatically reduced the ability of the *tor1Δ* cells to form colonies (Fig 8C), indicating that under extreme CR conditions, *TOR1*-regulated quiescence exit is mediated by all three kinases.

When left in the spent medium, CFU of the *tor1Δ* cells is significantly decreased by deletion of *RIM15* and marginally reduced by removal of *YAK1* (Fig 8D). Deletion of both *RIM15* and *YAK1* severely reduced the ability of the *tor1Δ* cells to form colonies. Within 12 days, almost all the *tor1Δrim15Δyak1Δ* cells lost their ability to exit from quiescence (Fig 8D), similar to the defects displayed by the *rim15Δyak1Δ* double mutants (Fig 4A). These data confirmed that the enhanced CFU enabled by *tor1Δ* deletion relies on Rim15 and Yak1. In contrast, deletion of *MCK1* reduced CFU of the *tor1Δ* cells only in the later stages of the assay (18 to 36 days, Fig 8D). This is in stark contrast to the similar CFU profiles exhibited by the *rim15Δ* and *mck1Δ* mutants in the WT background (Fig 4A). Furthermore, removal of *MCK1* did not further decrease but rather increased the CFU of the *tor1Δrim15Δ* or *tor1Δrim15Δyak1Δ* mutants (Fig 8D). Similarly, *MCK1* deletion increased the survival rate of the *tor1Δrim15Δ* cells in the early/mid stationary phase (Fig 8E), contrary to the significant decrease of cell survival observed when *MCK1* was removed from the *rim15Δ* mutant (Fig 8F). Previously, we have shown that the parallel relationship between *MCK1* and *RIM15* in the accumulation of storage carbohydrates is abrogated by *RAS2* deletion [21]. Put together, these data suggest that genetically compromising PKA or TOR may ameliorate the negative effects of *MCK1* deletion on quiescence exit. The mechanisms underlying these observations remain to be further elucidated. Nevertheless, both Rim15 and Mck1 are necessary for long-term survival of the *tor1Δ* cells in spent medium (>24 days; Fig 8E), supporting the conclusion that CLS extension enabled by *TOR1* deletion is regulated by the Rim15, Yak1 and Mck1 kinases (Fig 8C).

Relative CFU at day 24 displayed by the *tor1Δ* mutants in H_2_O (Fig 8C) correlated well with the amount of glycogen (R^2^ = 0.67, S6C Fig) or trehalose (R^2^ = 0.63, S6D Fig) but better with the sum of glycogen and trehalose (R^2^ = 0.78, S6E Fig), further supporting the contention that CLS extension is dependent on the accumulation of storage carbohydrates.

## Discussion

**Fig 9:**
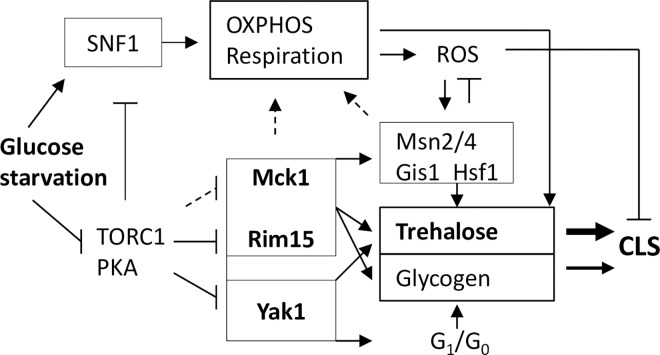
The working model showing that quiescence exit and chronological lifespan in yeast is dependent on the accumulation of storage carbohydrates and the intracellular ROS levels controlled by the kinases negatively regulated by TORC1/PKA. Yak1, Mck1 and Rim15 may also act together to negatively control the G_1_/S transition to limit the population entering the stationary phase. Arrow indicates activation; bar denotes inhibition and the dashed lines are regulations to be confirmed. Trehalose plays a dominant role over glycogen in CLS extension and therefore indicated by a broad arrow.

Our analyses of a diverse range of ‘signaling’ mutants and the single, double and triple mutants of *RIM15*, *YAK1* and *MCK1* indicate that deficiency in the accumulation of storage carbohydrates, rather than defects in the exit from the cell cycle, correlate well with the decrease of chronological lifespan (Fig 2, Fig 4 and Fig 8). Abolishing the accumulation of trehalose and glycogen dramatically reduces CLS (Fig 5). Overexpression of glycogen and trehalose biosynthetic genes, or addition of trehalose to the growth medium, rescues the defects of quiescence exit exhibited by the *rim15Δyak1Δ* and *rim15Δmck1Δ* mutants (Fig 6), further supporting the contention that the accumulation of trehalose and glycogen is essential to CLS extension. A positive correlation between storage carbohydrates and cell survival has been shown in chronologically aging cells [45]. Trehalose accumulation was associated with cellular proteostasis and CLS extension by calorie restriction or in *tor1Δ* and *sch9Δ* mutants [42,46]. Mutants unable to utilise glycogen or trehalose have significantly shortened CLS [46–47]. Put together, these and the data presented in this paper strongly support that the primary objective of the signaling pathways activated by glucose starvation is to reprogram carbon metabolism so that the transition-phase cells can accumulate sufficient energy stores to power quiescence exit (Fig 9). Trehalose seems to be a better predictor of CLS than glycogen in all but the *tor1Δ* mutants tested (Figs 2, 4, 5 and 8). These data suggest that the accumulation of trehalose plays a dominant role in the extension of CLS in cells bearing normal nutrient sensing pathways. However, glycogen storage makes a significant contribution to quiescence exit and long-term survival, especially when trehalose accumulation is compromised (Figs 5 and 9).

It is likely that the accumulation of storage carbohydrates coordinated by multiple pathways requires the metabolic reprogramming induced by glucose limitation and involves the activation of mitochondrial respiration and gluconeogenesis to convert the fermentation products to energy stores (Fig 9). Firstly, in batch cultures, glycogen synthesis starts when half of the initial glucose is consumed and peaks at the beginning of the diauxic shift [48]. Similarly induced are those genes involved in glycogen biosynthesis and *GAC1* [49], a positive regulator of glycogen synthase [50], which targets Glc7, the catalytic subunit of type-1 protein phosphatase, to activate Gsy2 by dephosphorylation [51–53]. Glycogen stores are partially utilised to fuel metabolic adaptations to respiratory growth and then refilled by the consumption of non-fermentative carbon sources, such as ethanol, acetate and glycerol [48,54–55]. Similarly, trehalose is accumulated during the diauxic and post-diauxic shift phases from glucose-derived fermentation products [48]. Respiratory mutants have been shown to be defective in the accumulation of storage carbohydrates and supplementation of trehalose rescues the severe CLS defects of these mutants [14]. Secondly, the SNF1 complex is a central regulator of respiration [56–57] and the mutants of the activators of the SNF1 complex or deletion of *SNF1* itself decreased the accumulation of energy stores and significantly reduced CLS (Table 1 and Fig 2). Thirdly, the Rim15/Gis1 pathway promotes the transcription of genes involved in the Krebs cycle, gluconeogenesis and the glyoxylate cycle [58–59]. The *rim15Δ* mutants have growth defects on non-fermentable carbon sources and they were outcompeted by WT cells in chemostat cultures limited for glucose or containing ethanol as the sole carbon source [59], indicating that the Rim15/Gis1 pathway is necessary for optimal respiratory growth (Fig 9). Furthermore, transcription of glycogen and trehalose biosynthetic genes is increased during the diauxic and post-diauxic-shift phases and this increase relies on Msn2/4 and Gis1 [60–61]. Phosphoproteomic studies have revealed that the phosphorylation status of Tsl1 and Gsy2 is regulated by Rim15 [18] and/or Mck1 [62–63]. Although the exact functions of these phosphorylations are yet to be revealed, these data suggest that these kinases may promote the accumulation of storage carbohydrates by stimulating mitochondrial respiration and gluconeogenesis to generate the substrates (UDP-Glc and Glu-6P) required for glycogen and trehalose synthesis and by modulating the relevant biosynthetic pathways at the transcriptional and post-transcriptional levels (Fig 9).

UDP-Glc is used for biosynthesis of both trehalose and glycogen. Compromising the trehalose synthesis enhanced the accumulation of glycogen (*tps1Δ*, Fig 5A), indicating that UDP-Glc used for trehalose synthesis is redirected towards glycogen accumulation. Glu-6P, the other substrate for trehalose synthesis, is an allosteric activator of glycogen synthase [64], suggesting that glycogen synthase activity could also be enhanced to increase glycogen accumulation when trehalose synthesis is compromised. It is intriguing to find that the Yak1 kinase has different roles in the accumulation of glycogen than Rim15 and Mck1 (Fig 3C). One possibility is that Yak1, Rim15 and Mck1 are all involved in the control of trehalose biosynthesis, but only Rim15 and Mck1 are implicated in glycogen accumulation (Fig 9). Given that Rim15 plays a more dominant role than Mck1 in glycogen accumulation (Fig 3C), the biosynthetic capacity directed towards glycogen in the *yak1Δ* mutant is more severely decreased in the *rim15Δ* than that in the *mck1Δ* cells, leading to lower levels of glycogen in the *rim15Δyak1Δ* than those in the *yak1Δmck1Δ* mutants (Fig 3C). Such a model, however, remains to be tested experimentally.

Cytotoxicity and cell death are often correlated with the levels of intracellular ROS, generated by mitochondrial respiration, ER stress, or as a result of peroxisomal dysfunction [65–66]. We have shown that the Rim15 and Yak1 act in parallel, and Mck1 has redundant function with Rim15 or Rim15 and Yak1 in their control of intracellular ROS (Fig 7A). Much higher levels of ROS were accumulated in the *rim15Δyak1Δ* than those in the *rim15Δmck1Δ* cells (Fig 7A). The *rim15Δyak1Δ* mutants lost their ability to exit from quiescence more quickly than the *rim15Δmck1Δ* cells in the WT (Fig 4A) or *tor1Δ* background (Fig 8D), even though the former mutants accumulated more storage carbohydrates than the latter cells (Figs 3C and 8B). These data suggest that the ability to exit quiescence may be dependent on the levels of storage carbohydrates as well as the amount of ROS accumulated in early stationary-phase cells. Trehalose supplementation restored the ability of the *rim15Δmck1Δ* mutants to exit from quiescence but only partially rescued the CFU defects of the *rim15Δyak1Δ* cells (Fig 6G and 6H). Trehalose accumulation *per se* did not greatly influence the levels of intracellular ROS (Fig 7B), further supporting that Rim15, Yak1 and Mck1 kinases regulate the accumulation of storage carbohydrates as well as the anti-oxidant defence systems to ensure long-term survival (Fig 9).

Previous transcript profiling indicated that starvation-induced anti-oxidant genes are upregulated by Rim15 through the activation of Gis1 and Msn2/4 [67]. In agreement with this, removal of *GIS1* and *MSN2/4* led to severe defects in response to oxidative stress (Fig 3B). It is likely that activation of such systems may also involve the Hsf1 transcription factor. First, we have demonstrated that expression of the pSSA3-RFP reporter is cooperatively regulated by Hsf1, Gis1 and Msn2/4 (S1 Fig). Others have shown that Hsf1 cooperates with Msn2/4 to induce the transcription of a number of starvation-induced stress genes [68–69]. Moreover, Hsf1 is confirmed as a member of the group of transcription activators that upregulate the oxidative stress-responsive genes [70]. Starvation-induced phosphorylation and activation of Gis1 and Msn2/4 are regulated by Rim15 and Yak1 [18,26,28]. Similarly, Hsf1 phosphorylation and activation has been shown to be dependent on Snf1, Yak1 and Rim15 kinases [26,28,71]. Although it is not clear how Mck1 activates starvation-induced gene expression, these data do support the idea that the activation of the stress response and the accumulation of storage carbohydrates are co-ordinately regulated by the same set of anti-aging pathways (Fig 9).

Yak1, Rim15 and Mck1 were also shown to control the population entering the stationary phase (Fig 3D). The significant size reduction in the unbudded (G_d_/G_1_) or budded (S-G_2_-M) cells (Fig 3E and 3F) and the merging of the G_d_ and G_1_ populations in the *rim15Δyak1Δ* or *rim15Δmck1Δ* FACS profiles ([21], S3 Fig) suggest that these kinases may limit population growth by negatively regulating the G_1_/S transition until the size threshold is reached (Fig 9). Rim15 was recently revealed as a negative regulator of the G_1_/S transition under nitrogen-starved or TORC1-inhibited conditions by stabilising Sic1, the inhibitor of S entry [72]. Upon nitrogen starvation, CdK Pho85 phosphorylates Hsp70 (Ssa3 is a member), triggers the displacement of the Ydj1 chaperone from Cln3 and promotes Cln3 degradation, thus delaying the onset of S phase [73]. These data suggest that starvation-activated signals may control cell size by regulating the G_1_/S transition directly or indirectly through the activation of the stress response. Future work should aim to reveal the molecular mechanisms by which these signals coordinate metabolic reprogramming, stress response and the cell cycle during the transition into quiescence (Fig 9). Metabolic disorders and cell cycle dysregulations lie at the heart of many age-related diseases, such as diabetes and cancer. Revealing these mechanisms will enhance our understanding of the aging process in yeast and age-related diseases in mammals [1].

## Materials and Methods

### Strains and plasmids

Strains carrying single-gene deletions were obtained directly from the BY4742 mutant library (Open Biosystems). Strains carrying deletions in multiple genes were generated by combining mutations by either mating and tetrad dissection, or PCR-mediated gene replacement using drug resistant or nutritional markers [74–75]. Deletion mutants of genes coding for non-essential proteins involved in signaling pathways were selected based on data in Lee *et al*. [22]. Expression reporter cassettes were described previously [21]. *GSY2* under the control of its endogenous promoter was cloned into pRS425 for overexpression studies. Similarly, *TPS1* or *TSL1* controlled by their own promoters was cloned into pRS426. Mutations of the heat shock element in the *SSA3* promoter (in the pSSA3-RFP construct) were introduced by using a site-directed mutagenesis kit following the protocols described by the manufacturer (Stratagene). The second repeat of the NGAAN sequence of the HSE (5’-NGAANN_5_NGAANN_5_NGAAN-3’) was replaced by NTTAN. The new plasmid was named pSSA3(HSEΔ)-RFP (S1 Fig).

### Reporter fluorescence detection and quantification

WT (BY4742) and mutant cells bearing the pSSA3-RFP constructs were grown overnight in SMM liquid medium [76] containing 2% glucose, arrayed in quadruplicates onto SMM agar plates containing 0.6% glucose using Rotor HAD (Singer Instruments) and incubated for 3 days before imaging. CellProfiler [77] was used to determine the relative levels of RFP in WT and mutants. For quantitative assays of pSSA3-RFP and pHSP12-HSP12-VFP levels in liquid cultures, freshly-grown overnight cultures were inoculated (5% v/v) into SMM medium containing 0.6% glucose which had been dispensed into 96-well microtiter plate. Cell density (OD_595nm_) and fluorescence intensities were simultaneously monitored in triplicate using a plate reader (BMG Biotech). RFP is excited at 580/±10nm and emits at 610/±10nm, while VFP is excited at 500/±10nm and emits at 540/±10nm. Medium-only blanks and WT cells bearing the same constructs were included for each run as negative and positive controls. After background subtraction, RFP and VFP fluorescence intensities were normalized to cell density (OD_595nm_). The mean and standard deviation from triplicates (Fig 1A) were calculated at each time point; for the sake of clarity, standard errors were not plotted with the means in Fig 1B.

### Determination of storage carbohydrates

The concentrations of glycogen and trehalose (μg glucose per mg of wet cells) in early stationary-phase cells was determined, following the procedures described by Parrou and Francois [78] as described previously [21].

### Phenotypic assays

Stress resistance conferred by cells grown to early stationary phase (after 3 days incubation in YPD (2% glucose) or buffered SC medium [41]) was determined as described previously [21]. Yeast cells were subjected to treatment at 55°C for 5 or 10 minutes, serially diluted and spotted onto YPD agar to determine their resistance to heat shock. Similarly, cells were directly spotted onto YPD agar plate containing 2.5mM or 5mM H_2_O_2_ to assay their resistance to oxidative stress.

### CLS assays

Two different CLS measurements were adopted throughout the experiments. One was to assess the ability of stationary-phase cells to exit from quiescence to form colonies and the other to determine the viability of individual cells in the population during the stationary phase. The ability of quiescent cells to form colonies was measured every three, six or seven days by normalising colony-forming units (CFU) of stationary-phase cultures to that produced by the day 0 culture, which is early stationary phase culture after 3 days of growth in YPD (2% glucose) [21] or buffered SC medium containing 2% glucose [79]. The number of CFU is scored by spreading cells onto YPD plates after serial dilutions (1:20/1:50/1:100 for cultures grown in YPD or 1:10/1:50/1:50 for cultures grown in buffered SC medium). Cell viability was determined by FACS analysis, essentially following the protocol described by Ocampo and Barrientos [80], using 2μM of Sytox Green instead of propidium iodide. Excitation was performed using a laser at 488nm and emission detected with a standard 530/30 band pass filter.

### Analysis of cell cycle and budding index

Cell cycle status was determined according to Haase and Reed [81] using Sytox green to stain DNA in fixed cells. Samples were sonicated at low power (2 min) and analyzed using a cytometer (LSRFortessa, Becton Dickinson). Data were processed and the percentage of budded cells (S-G_2_-M) and cells carrying 3C DNA analyzed using FlowJo software (www.flowjo.com).

### FACS analysis of intracellular Reactive Oxygen Species

Levels of intracellular ROS in live cells were determined by flow cytometry after dihydrorhodamine 123 (DHR) staining according to established procedures [82]. Briefly, 1 × 10^7^ cells were harvested by centrifugation for 1 min at 13,000rpm at room temperature; washed once with 200 μl of PBS and then resuspended in 200 μl of PBS. Aliquotes of cells (50 μl) were added to 1ml PBS containing 10 μM of DHR (diluted from 10 mM stock in ethanol, Sigma-Aldrich). Following incubation in the dark at 30°C for 60 min, the cells were harvested, washed once with 200 μl of PBS and resuspended in 1ml of PBS. After mild sonication, cells are then analyzed by flow cytometry (488nm, 530/30). Data collected from flow cytometer were processed with Flowjo software.

## Supporting Information

S1 Fig Starvation-induced pSSA3-RFP expression is regulated by Hsf1, Msn2/4 and Gis1. 1a: The relative levels of pSSA3-RFP in WT (BY4741) and DAmP^hsf1^ cells; 1b: The relative levels of pSSA3-RFP and pSSA3(HSEΔ)-RFP in WT (BY4742) and *msn2/4Δgis1Δ* triple mutants.
(PPTX)

S2 Fig FACS profiles of WT, *sbp1Δ*, *sak1Δ* and *rhr2Δ* cells grown in YPD for 11h (top) and 24h (bottom). Cells encircled in red have 3C DNA.
(PPTX)

S3 Fig FACS profiles of WT, the single, double and triple deletants of *rim15Δ*, *yak1Δ* and *mck1Δ* during the transition into the stationary phase. At 12h, glucose is consumed in all cultures except for the *yak1Δmck1Δ* mutant, in which the glucose concentration is approximately 0.2%. The profiles of WT, *rim15Δ*, *mck1Δ* and *rim15Δmck1Δ* mutants [21] were included here for cross comparison.
(PPTX)

S4 Fig Chronological lifespan is closely correlated with storage carbohydrates accumulated in early stationary-phase cells. **4a** and **4b**: Relative CFU at day 12 (4a) and day 18 (4b) is highly correlated with storage carbohydrates accumulated in WT, the single, double and triple mutants of *YAK1*, *RIM15* and *MCK1*. **4c**, **4d** and **4e**: Relative CFU at day 14 correlates poorly with glycogen, very well with trehalose, but better with total amount of carbohydrates accumulated in WT and mutants of trehalose/glycogen biosynthesis.
(PPTX)

S5 Fig Cell viability of WT (5a), *rim15Δyak1Δ* (5b) and *rim15Δmck1Δ* (5c) mutants bearing the empty vectors (pRS425/pRS426), *GSY2*/*TPS1* or *GSY2/TSL1* expression constructs.
(PPTX)

S6 Fig *TOR1* deletion does not abrogate the correlation between CLS and storage carbohydrates. **6a** and **6b**: Deletion of *TOR1* in BY4742 mildly enhances CLS in the first 30 days in H_2_O (6a) or in spent medium (6b). **6c**, **6d** and **6e**: Enhanced CLS by *tor1Δ* deletion is mediated by *YAK1*, *RIM15* and *MCK1*, and correlated well with glycogen (6c), trehalose (6d) but best with total storage carbohydrates (6e) accumulated in early stationary-phase cells.
(PPTX)
