# Supplementary material for: Chronological Lifespan in Yeast Is Dependent on the Accumulation of Storage Carbohydrates Mediated by Yak1, Mck1 and Rim15 Kinases
Source: PLoS Genet. 2016 Dec 6;12(12):e1006458. doi: 10.1371/journal.pgen.1006458 (PMC5140051; doi:10.1371/journal.pgen.1006458)
Supplement: S1 Fig — 1a: The relative levels of pSSA3-RFP in WT (BY4741) and DAmPhsf1 cells; 1b: The relative levels of pSSA3-RFP and pSSA3(HSEΔ)-RFP in WT (BY4742) and msn2/4Δgis1Δ triple mutants. (PPTX) [file pgen.1006458.s001.pptx]

## Slide 1
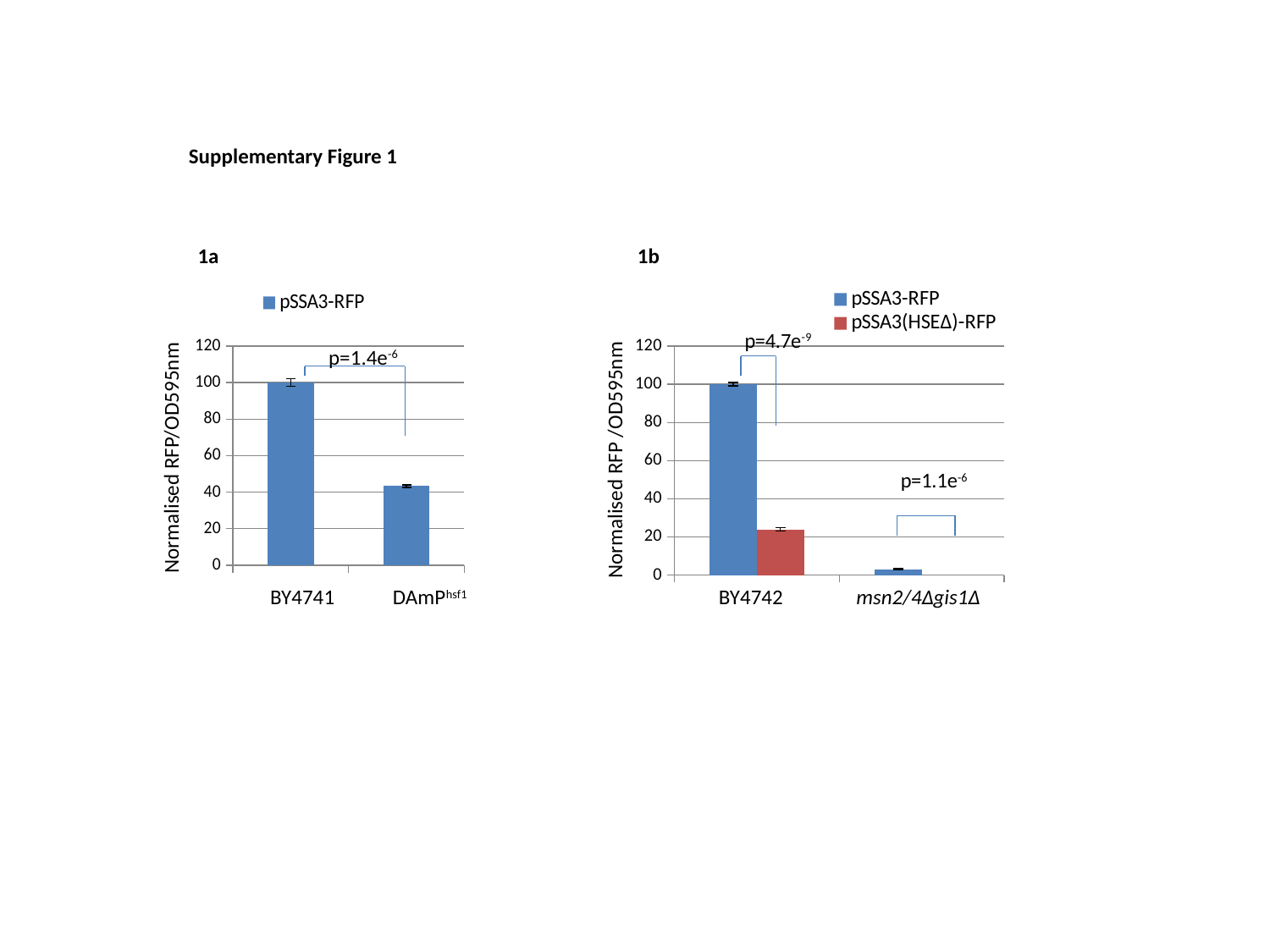

Supplementary Figure 1
1a
1b
### Chart
| Category | |
|---|---|
### Chart
| Category | | |
|---|---|---|p=1.4e-6
Normalised RFP/OD595nm
Normalised RFP /OD595nm
BY4741 DAmPhsf1
BY4742 msn2/4∆gis1∆
