# Supplementary figures and images for: Chronological Lifespan in Yeast Is Dependent on the Accumulation of Storage Carbohydrates Mediated by Yak1, Mck1 and Rim15 Kinases
Source: PLoS Genet. 2016 Dec 6;12(12):e1006458. doi: 10.1371/journal.pgen.1006458 (PMC5140051; doi:10.1371/journal.pgen.1006458)

## Slide 1
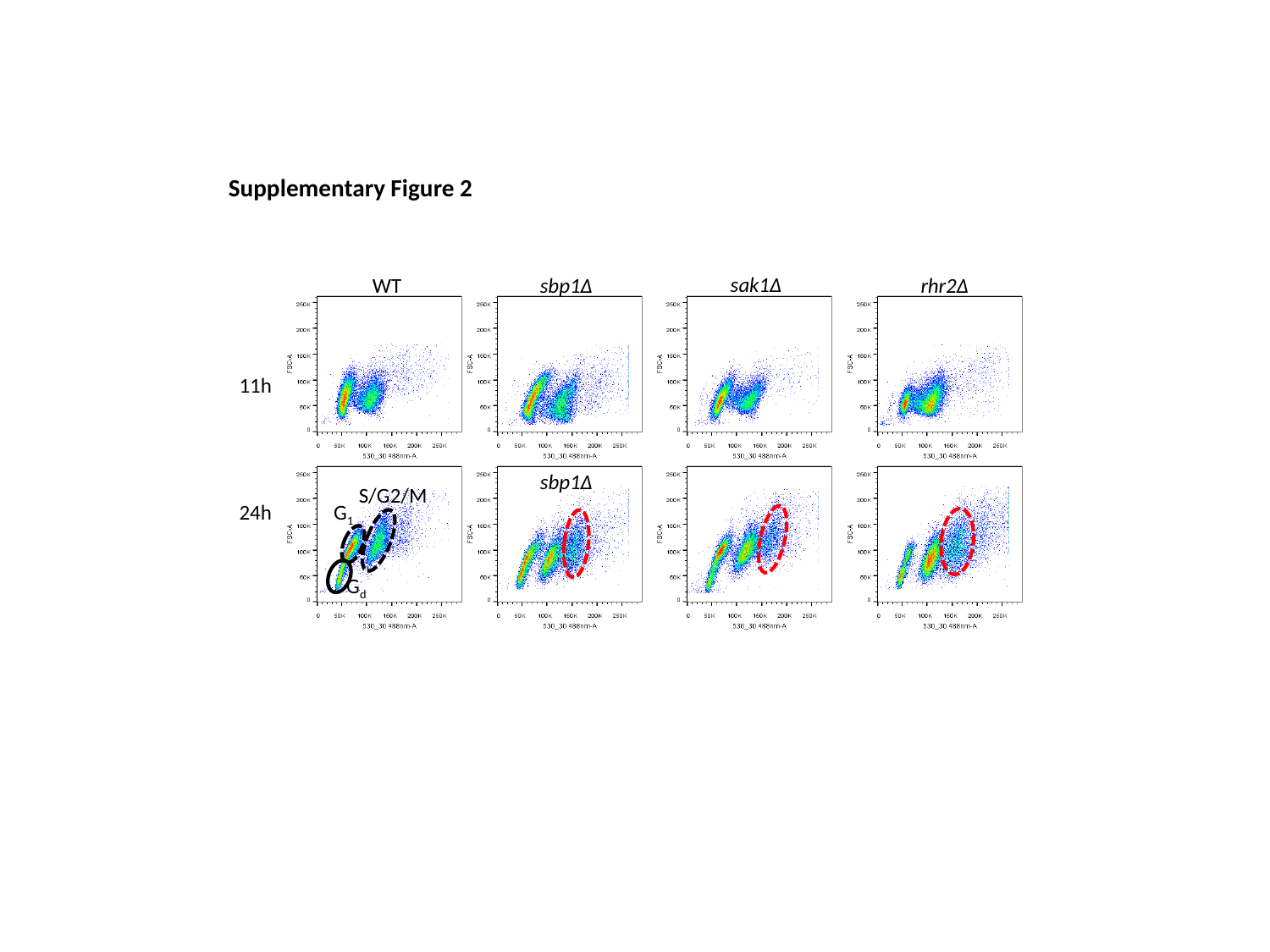

Supplementary Figure 2
sak1∆
WT
sbp1∆
rhr2∆
11h
24h
sbp1∆
S/G2/M
G1
Gd

Supplement: S2 Fig — Cells encircled in red have 3C DNA. (PPTX) [file pgen.1006458.s002.pptx]

## Slide 1
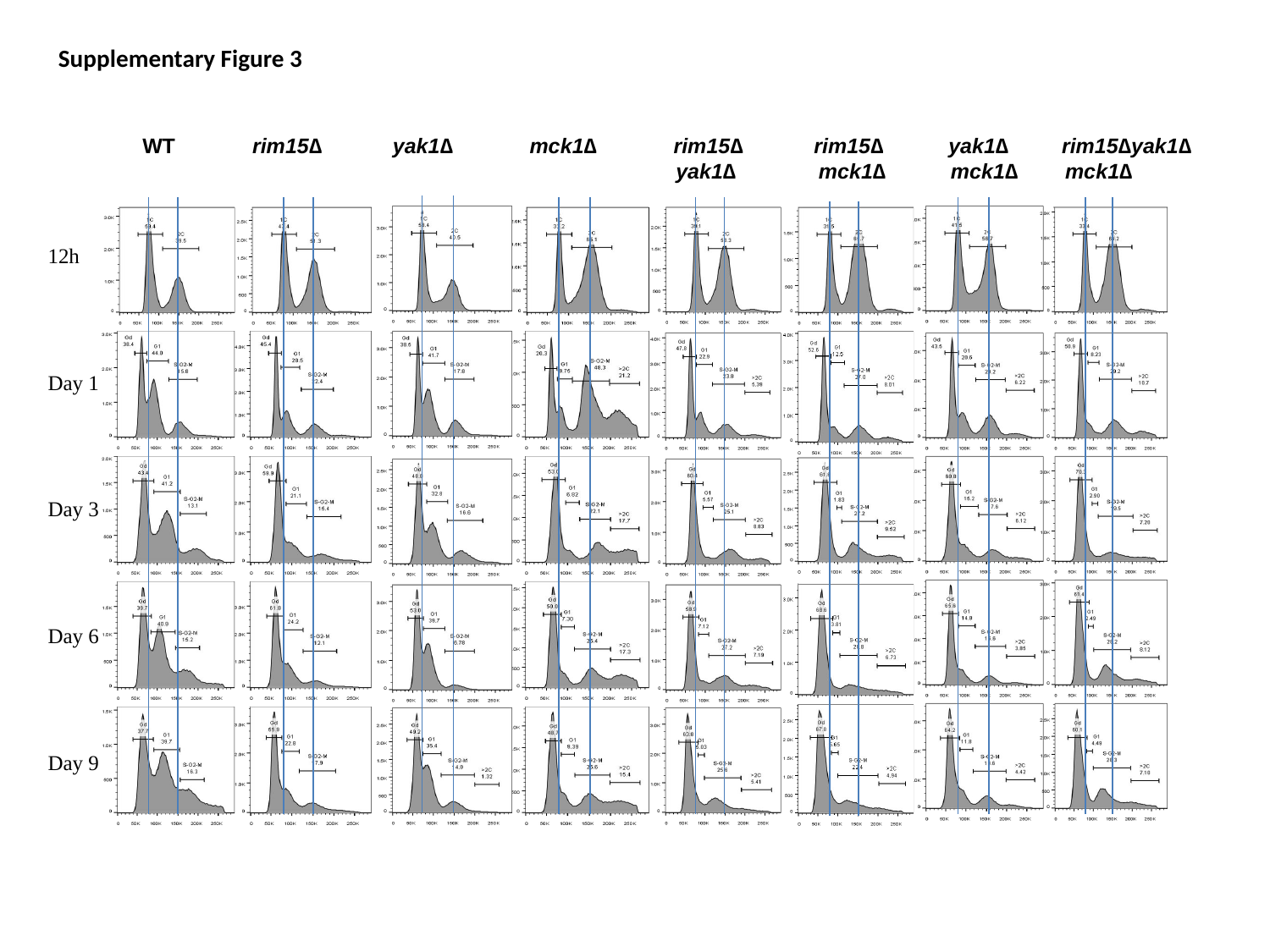

Supplementary Figure 3
WT rim15∆ yak1∆ mck1∆ rim15∆ rim15∆ yak1∆ rim15∆yak1∆
 yak1∆ mck1∆ mck1∆ mck1∆
12h
Day 1
Day 3
Day 6
Day 9

Supplement: S3 Fig — At 12h, glucose is consumed in all cultures except for the yak1Δmck1Δ mutant, in which the glucose concentration is approximately 0.2%. The profiles of WT, rim15Δ, mck1Δ and rim15Δmck1Δ mutants [21] were included here for cross comparison. (PPTX) [file pgen.1006458.s003.pptx]
