# Supplementary material for: Chronological Lifespan in Yeast Is Dependent on the Accumulation of Storage Carbohydrates Mediated by Yak1, Mck1 and Rim15 Kinases
Source: PLoS Genet. 2016 Dec 6;12(12):e1006458. doi: 10.1371/journal.pgen.1006458 (PMC5140051; doi:10.1371/journal.pgen.1006458)
Supplement: S4 Fig — 4a and 4b: Relative CFU at day 12 (4a) and day 18 (4b) is highly correlated with storage carbohydrates accumulated in WT, the single, double and triple mutants of YAK1, RIM15 and MCK1. 4c, 4d and 4e: Relative CFU at day 14 correlates poorly with glycogen, very well with trehalose, but better with total amount of carbohydrates accumulated in WT and mutants of trehalose/glycogen biosynthesis. (PPTX) [file pgen.1006458.s004.pptx]

## Slide 1
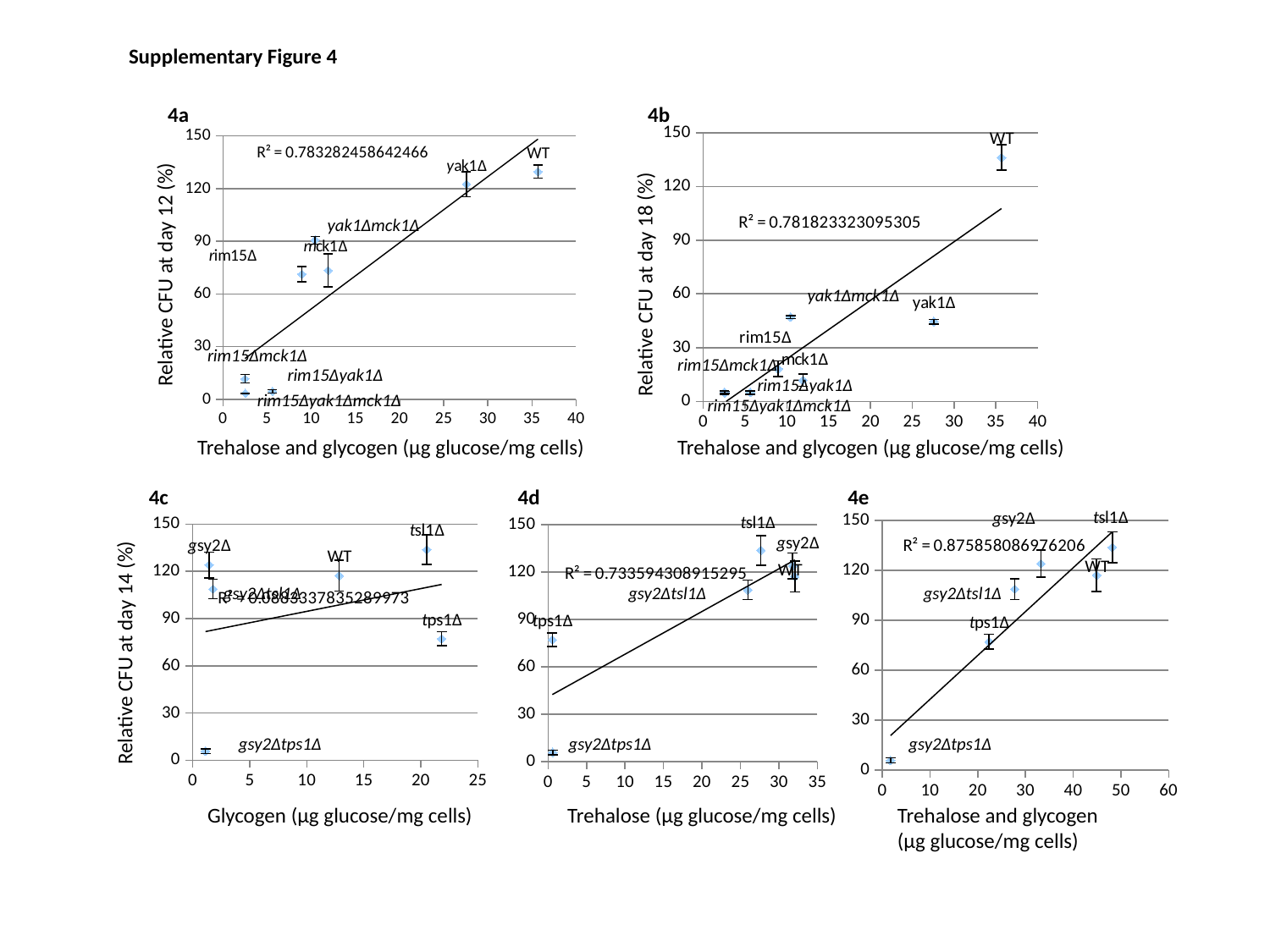

Supplementary Figure 4
4a
4b
### Chart
| Category | |
|---|---|
### Chart
| Category | |
|---|---|yak1∆mck1∆
Relative CFU at day 12 (%)
Relative CFU at day 18 (%)
yak1∆mck1∆
rim15∆mck1∆
rim15∆mck1∆
rim15∆yak1∆
rim15∆yak1∆
rim15∆yak1∆mck1∆
rim15∆yak1∆mck1∆
Trehalose and glycogen (µg glucose/mg cells)
Trehalose and glycogen (µg glucose/mg cells)
4c
4d
4e
### Chart
| Category | |
|---|---|
### Chart
| Category | |
|---|---|
### Chart
| Category | |
|---|---|gsy2∆tsl1∆
gsy2∆tsl1∆
gsy2∆tsl1∆
Relative CFU at day 14 (%)
gsy2∆tps1∆
gsy2∆tps1∆
gsy2∆tps1∆
Glycogen (µg glucose/mg cells)
Trehalose (µg glucose/mg cells)
Trehalose and glycogen
(µg glucose/mg cells)
