# Supplementary material for: Chronological Lifespan in Yeast Is Dependent on the Accumulation of Storage Carbohydrates Mediated by Yak1, Mck1 and Rim15 Kinases
Source: PLoS Genet. 2016 Dec 6;12(12):e1006458. doi: 10.1371/journal.pgen.1006458 (PMC5140051; doi:10.1371/journal.pgen.1006458)
Supplement: S5 Fig — Cell viability of WT (5a), rim15Δyak1Δ (5b) and rim15Δmck1Δ (5c) mutants bearing the empty vectors (pRS425/pRS426), GSY2/TPS1 or GSY2/TSL1 expression constructs. (PPTX) [file pgen.1006458.s005.pptx]

## Slide 1
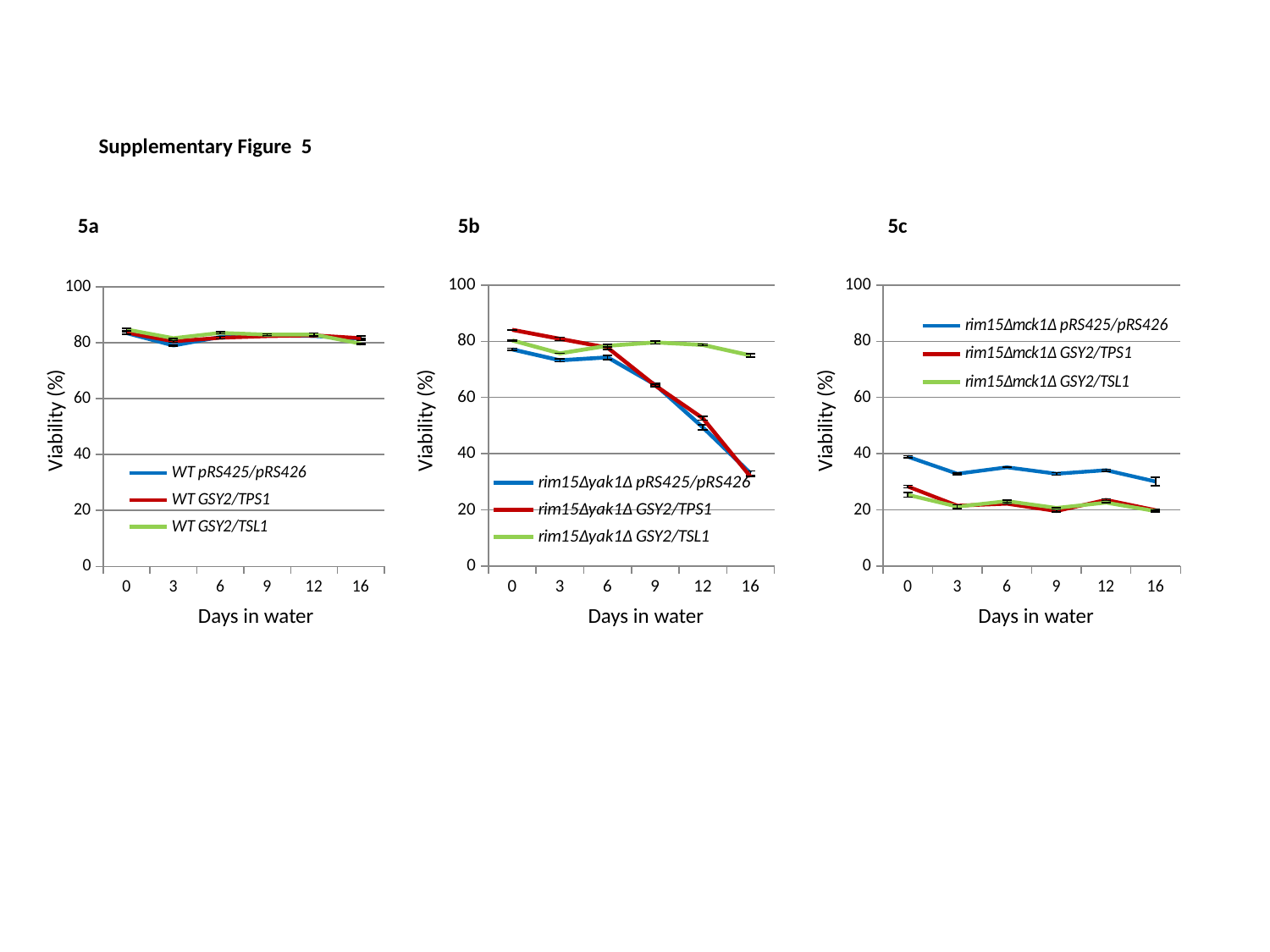

Supplementary Figure 5
5a
5b
5c
### Chart
| Category | | | |
|---|---|---|---|
| 0 | 83.39999999999999 | 83.60000000000001 | 84.7 |
| 3 | 78.9666666666667 | 80.4666666666667 | 81.6 |
| 6 | 82.0 | 81.73333333333325 | 83.50000000000001 |
| 9 | 82.53333333333329 | 82.3 | 82.86666666666667 |
| 12 | 82.36666666666667 | 82.63333333333316 | 82.89999999999999 |
| 16 | 81.36666666666667 | 81.60000000000001 | 79.63333333333317 |
### Chart
| Category | | | |
|---|---|---|---|
| 0 | 77.10000000000001 | 84.06666666666668 | 80.3 |
| 3 | 73.23333333333325 | 80.86666666666666 | 75.7 |
| 6 | 74.26666666666667 | 77.73333333333325 | 78.4 |
| 9 | 64.56666666666666 | 64.26666666666667 | 79.60000000000001 |
| 12 | 49.3666666666665 | 52.6 | 78.7 |
| 16 | 33.0 | 31.8 | 74.9666666666667 |
### Chart
| Category | | | |
|---|---|---|---|
| 0 | 38.9 | 28.3 | 25.333333333333275 |
| 3 | 32.833333333333336 | 21.46666666666667 | 21.133333333333283 |
| 6 | 35.13333333333333 | 22.166666666666668 | 23.099999999999987 |
| 9 | 32.833333333333336 | 19.566666666666666 | 20.666666666666668 |
| 12 | 34.1 | 23.533333333333264 | 22.566666666666666 |
| 16 | 30.066666666666666 | 19.866666666666667 | 19.533333333333264 |Viability (%)
Viability (%)
Viability (%)
Days in water
Days in water
Days in water
