# Supplementary material for: Chronological Lifespan in Yeast Is Dependent on the Accumulation of Storage Carbohydrates Mediated by Yak1, Mck1 and Rim15 Kinases
Source: PLoS Genet. 2016 Dec 6;12(12):e1006458. doi: 10.1371/journal.pgen.1006458 (PMC5140051; doi:10.1371/journal.pgen.1006458)
Supplement: S6 Fig — 6a and 6b: Deletion of TOR1 in BY4742 mildly enhances CLS in the first 30 days in H2O (6a) or in spent medium (6b). 6c, 6d and 6e: Enhanced CLS by tor1Δ deletion is mediated by YAK1, RIM15 and MCK1, and correlated well with glycogen (6c), trehalose (6d) but best with total storage carbohydrates (6e) accumulated in early stationary-phase cells. (PPTX) [file pgen.1006458.s006.pptx]

## Slide 1
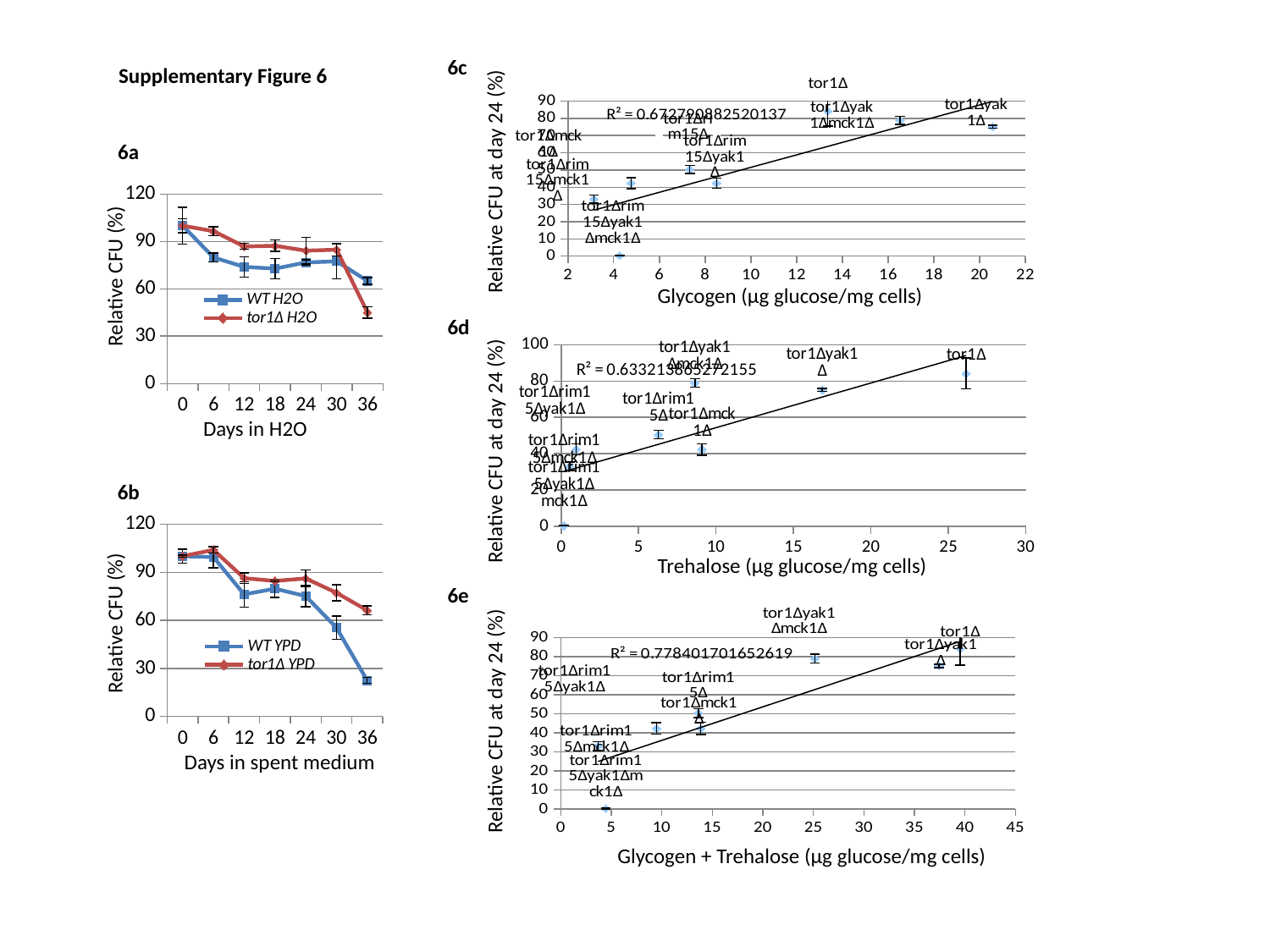

6c
Supplementary Figure 6
### Chart
| Category | |
|---|---|6a
Relative CFU at day 24 (%)
### Chart
| Category | | |
|---|---|---|
| 0 | 100.0 | 100.0 |
| 6 | 79.89323843416368 | 96.50205761316869 |
| 12 | 73.84341637010675 | 86.83127572016461 |
| 18 | 72.7758007117435 | 87.24279835390902 |
| 24 | 76.69039145907472 | 84.1563786008226 |
| 30 | 77.40213523131672 | 84.77366255144031 |
| 36 | 64.94661921708186 | 45.06172839506176 |Days in H2O
Relative CFU (%)
Glycogen (µg glucose/mg cells)
6d
### Chart
| Category | |
|---|---|Relative CFU at day 24 (%)
6b
### Chart
| Category | | |
|---|---|---|
| 0 | 100.0 | 100.0 |
| 6 | 99.4661921708185 | 103.9451114922813 |
| 12 | 76.15658362989323 | 86.27787307032588 |
| 18 | 79.7153024911032 | 84.56260720411663 |
| 24 | 75.08896797153025 | 86.10634648370466 |
| 30 | 55.33807829181494 | 77.18696397941682 |
| 36 | 22.41992882562279 | 66.20926243567781 |Days in spent medium
Trehalose (µg glucose/mg cells)
6e
Relative CFU (%)
### Chart
| Category | |
|---|---|Relative CFU at day 24 (%)
Glycogen + Trehalose (µg glucose/mg cells)
